# HIV Drug Resistance in Adults Initiating or Reinitiating Antiretroviral Therapy in Uruguay—Results of a Nationally Representative Survey, 2018–2019

**DOI:** 10.3390/v15020490

**Published:** 2023-02-10

**Authors:** Rosa Flieller, Susana Cabrera, Dora Ruchansky, Amalia Girón-Callejas, María Brasesco, Daniel Pérez, Héctor Chiparelli, Claudia García-Morales, Daniela Tapia-Trejo, Jessica Monreal-Flores, Giovanni Ravasi, Michael R. Jordan, Santiago Ávila-Ríos

**Affiliations:** 1 Unidad de Virología, Departamento de Laboratorios de Salud Pública, Ministerio de Salud Pública, Montevideo 11600, Uruguay; 2 Programa de ITS-VIH/SIDA, Ministerio de Salud Pública, Montevideo 11200, Uruguay; 3 Facultad de Medicina, Universidad de la República, Montevideo 11800, Uruguay; 4 Centre for Research in Infectious Diseases, National Institute of Respiratory Diseases, Mexico City 14080, Mexico; 5 Pan American Health Organization, Washington, DC 20037, USA; 6 Division of Geographic Medicine and Infectious Diseases, Tufts Medical Center, Boston, MA 02111, USA; 7 Levy Center for Integrated Management of Antimicrobial Resistance, Tufts University School of Medicine, Boston, MA 02111, USA

**Keywords:** drug resistance surveillance, Uruguay, HIV

## Abstract

The first nationally representative cross-sectional HIV drug resistance (HIVDR) survey was conducted in Uruguay in 2018–2019 among adults diagnosed with HIV and initiating or reinitiating antiretroviral therapy (ART). *Protease*, *reverse transcriptase*, and *integrase* genes of HIV-1 were sequenced. A total of 206 participants were enrolled in the survey; 63.2% were men, 85.7% were >25 years of age, and 35.6% reported previous exposure to antiretroviral (ARV) drugs. The prevalence of HIVDR to efavirenz or nevirapine was significantly higher (OR: 1.82, *p* < 0.001) in adults with previous ARV drug exposure (20.3%, 95% CI: 18.7–22.0%) compared to adults without previous ARV drug exposure (12.3%, 11.0–13.8%). HIVDR to any nucleoside reverse transcriptase inhibitors was 10.3% (9.4–11.2%). HIVDR to ritonavir-boosted protease inhibitors was 1.5% (1.1–2.1%); resistance to ritonavir-boosted darunavir was 0.9% (0.4–2.1%) among adults without previous ARV drug exposure and it was not observed among adults with previous ARV drug exposure. Resistance to integrase inhibitors was 12.7% (11.7–13.8%), yet HIVDR to dolutegravir, bictegravir, and cabotegravir was not observed. The high level (>10%) of HIVDR to efavirenz highlights the need to accelerate the transition to the WHO-recommended dolutegravir-based ART. Access to dolutegravir-based ART should be prioritised for people reporting previous ARV drug exposure.

## 1. Introduction

HIV drug resistance negatively impacts the effectiveness of antiretroviral (ARV) drugs in preventing and treating HIV infection. HIV drug resistance increases the number of HIV/AIDS-associated deaths, new HIV infections, and antiretroviral therapy (ART) programme costs [1]. Therefore, the surveillance, prevention and control of HIV drug resistance are essential to achieving the 95-95-95 targets and the elimination of AIDS as a public health threat by 2030 [2]. The World Health Organization (WHO) recommends the surveillance of HIV drug resistance among adults initiating and reinitiating ART to inform the selection of effective first-line ART regimens and adequate prophylaxis regimens [3].

The prevalence of HIV drug resistance among adults initiating and reinitiating ART has increased in Latin America and the Caribbean: An annual increase of 11% (95% CI: 5–18%) in the odds of HIV drug resistance to non-nucleoside reverse transcriptase inhibitors (NNRTI) was reported from 2007 to 2016 [4]. Furthermore, significantly higher levels of NNRTI resistance (OR: 3.4, 95% CI: 1.8–6.9%, *p* < 0.0001) have been reported among adults with previous ARV drug exposure compared with those without previous ARV drug exposure [4]. Several low- and middle-income countries have reported a nationally representative prevalence of HIV drug resistance to efavirenz (EFV) or nevirapine (NVP) above 10% among adults initiating and reinitiating ART [5], the WHO-recommended threshold to urge transition to a non-NNRTI-containing regimen [6].

This report presents the findings of the first nationally representative survey to estimate the prevalence of HIV drug resistance among adults initiating and reinitiating ART in Uruguay from October 2018 to October 2019. At the time of the survey, 9240 adults living with HIV were receiving ART, and <1000 adults were newly infected with HIV annually [7].

## 2. Materials and Methods

### 2.1. Study Design

The Ministry of Health of Uruguay implemented a cross-sectional nationally representative survey following the WHO-recommended methods [3]. Twenty-two public and private clinics provided ART in Uruguay at the time of the survey. Eleven ART clinics were selected as survey sites. These clinics served 94% of all adults initiating and reinitiating ART in Uruguay. The excluded eleven ART clinics had less than ten adults initiating and reinitiating ART per year. The sample size was calculated using the WHO sample size calculator tool and utilised a finite population correction [8]. The following assumptions were used to calculate the sample size: (a) a prevalence of HIV drug resistance to EVF or NVP of 10% among adults initiating and reinitiating ART, (b) an absolute precision of 5%, (c) a genotyping failure rate of 20%, (d) 840 adults initiating and reinitiating ART in 2016, (e) a proportion of previous ARV drug exposure of 25%, and (f) a proportion of adults initiating and reinitiating ART with regimens that included NNRTIs of 80%. Using these assumptions, the total sample size was 239, and it was distributed among survey sites proportionally to the number of adults initiating or reinitiating ART at each of the eleven clinics selected to participate in the survey.

### 2.2. Participant Enrolment

Adults ≥ 18 years of age diagnosed with HIV initiating or reinitiating ART were eligible for survey enrolment. Eligible individuals who provided verbal informed consent were consecutively enrolled. Deidentified demographic and clinical data were abstracted from clinical records. Previous ARV drug exposure was classified as pre-exposure prophylaxis, post-exposure prophylaxis, prevention of mother-to-child transmission, discontinuation of prior ART, or a combination of these exposures.

### 2.3. Laboratory Procedures

Whole blood specimens were collected by venipuncture in EDTA-containing tubes from participants before initiating or reinitiating ART. Plasma was separated within 12 h of collection; all plasma specimens were stored at −80 °C at the National HIV-AIDS Reference Laboratory of Uruguay until the completion of the specimen collection period (October 2019). Plasma specimens were shipped on dry ice to the Centre for Research in Infectious Diseases of the National Institute of Respiratory Diseases in Mexico City, a WHO-designated regional laboratory for HIV drug resistance testing.

HIV RNA was extracted from 1 mL of plasma (QIAamp Viral RNA Kit; QIAGEN, Valencia, CA, USA). *Protease* (HXB2 positions 6–99), *reverse transcriptase* (HXB2 positions 1–251), and *integrase* (HXB2 positions 1–288) HIV genes were amplified and sequenced using in-house-validated protocols [9,10,11]. Sanger sequencing was performed on a 3730xl Genetic Analyser (ThermoFisher, Waltham, MA, USA). Sequences were assembled using ReCall [12], and the post-testing quality assurance was carried out using the WHO/BCCfE HIVDR quality control tool [13]. The genotyping success rate was 99.0% (204/206) for *protease* and *reverse transcriptase* and 99.5% (205/206) for *integrase*.

### 2.4. Data Analysis

Five pairs of HIV sequences with a small genetic distance (<0.5%) and without epidemiological link were detected. Therefore, following the WHO/HIV ResNet Laboratory Operational Framework [14], one sequence was excluded from each pair. Thus, 204 reverse transcriptase and protease sequences and 205 integrase sequences were used for the analysis. HIV drug resistance was predicted using the Stanford HIV drug resistance database (HIVdb v 8.9-1) tool [15,16]. Sequences with a penalty score ≥ 15 were considered resistant to a given ARV drug. HIV subtype was assigned using the REGA HIV-1 subtyping tool (v3.46) [17].

Weighted statistical analysis was performed according to WHO recommendations [3], using STATA 15.1 (StataCorp, College Station, TX, USA). The weights were calculated based on the estimated eligible population and the number of individuals enrolled in each survey site. The analysis of HIV drug resistance was weighted considering the genotyping success rate. Odds ratios were calculated by logistic regression accounting for survey design and weights. Statistical significance was assessed at the 0.05 level.

An HIV transmission network analysis was performed to identify and characterise transmission clusters. Sequences were aligned by codons using Mega v11 [18]. Phylogenetic trees were constructed using the maximum likelihood method based on the General Time Reversible model with 1000 bootstrap repetitions, including reference sequences for HIV subtypes obtained from the Los Alamos HIV Sequences Database (www.hiv.lanl.gov, accessed on 2 August 2022). The trees were visualised and coloured in Mega v11 [18]. Putative transmission links were defined and resolved between individuals whose HIV sequences had a genetic distance <1.5% using HIV-TRACE [19].

## 3. Results

### 3.1. Demographic and Clinical Characteristics

From October 2018 to October 2019, 206 adults who initiated or reinitiated ART in Uruguay were enrolled in the survey; 63.2% (95% CI: 62.1–64.3%) were men, and 85.7% (95% CI: 85.2–86.2%) were over 25 years of age (Table 1). All individuals enrolled in the study had an HIV viral load >1000 copies/mL. Among those with a CD4 lymphocyte count result available at the time of treatment initiation (65%), 28.9% (IC95% 24–34.2%) had <200 cells/µL, 39.2% (IC95% 34.2–44.4%) had between 200 and 500 cells/µL, and 32.0% (IC95% 27.2–37.1%) had >500 cells/µL. The proportion of individuals with CD4 lymphocyte count <200 cells/µL was similar among individuals who reported previous exposure to ARV drugs (29.0%, IC95% 24.5–33.8%) and those without previous ARV drug exposure (28.8%, IC95% 23.2–35.1%).

The proportion of individuals who reported previous exposure to ARV drugs was 35.6% (95% CI: 34.2–37.0%). Men were less likely to have been exposed to ARV drugs before survey enrolment than women (*p* < 0.001). People older than 25 years of age were more likely to have had previous ARV drug exposure(s) than people 25 years of age or younger (*p* < 0.001). Prior ART for the treatment of HIV infection followed by treatment interruption of >3 months was the most commonly reported type of previous ARV drug exposure (79.8%, 95% CI: 69.8–87.0%%, n/N: 58/73), followed by post-exposure prophylaxis (1.3%, 95% CI: 0.2–10.7%, n/N: 1/73), and prevention of mother-to-child transmission of HIV (1.3%, 95% CI: 0.2–10.6%%, n/N: 1/73). The type of previous exposure to ARV drugs was unknown in 17.6% (95% CI: 11.3–26.4%%, n/N: 13/73) of the cases.

The most frequently observed HIV-1 subtypes were subtype B (42.0%), F1/B recombinants (25.4%), and subtype F (8.3%) (Table 2).

### 3.2. HIV Drug Resistance

The prevalence of HIV drug resistance to EFV or NVP was 15.2% (95% CI: 14.1–16.3%), 10.3% (95% CI: 9.4–11.2%) to any nucleoside reverse transcriptase inhibitor, 12.7% (95% CI: 11.7–13.8%) to integrase strand transfer inhibitor (INSTI), and 1.5% (95% CI: 1.1–2.1%) to boosted protease inhibitors (PI/r). INSTI resistance was attributed to the first-generation INSTI (elvitegravir and raltegravir). Resistance to dolutegravir, bictegravir, and cabotegravir was not observed (Table 3).

The prevalence of HIV drug resistance to EFV or NVP was significantly higher (OR: 1.82, 95% CI: 1.53–2.16, *p* < 0.001) in adults with previous exposure to ARV drugs (20.3%, 95% CI: 18.7–22.0%) compared to adults without previous exposure to ARV drugs (12.3%, 95% CI: 11.0–13.8%) (Table 3). Among people who reported previous ARV drug exposure, men were significantly more likely to have HIV drug resistance to EFV or NVP than women (OR: 1.50, 95% CI: 1.15–1.96, *p* = 0.005).

The prevalence of HIV drug resistance to tenofovir disoproxil fumarate (TDF) was 3.1% (95% CI: 2.4–4.1%); no HIV drug resistance to emtricitabine (FTC) or lamivudine (3TC) was observed among adults without previous ARV drug exposure.

Resistance to the first-generation INSTIs was 14.9% (95% CI: 14.1–15.6%) in adults without previous exposure to ARV drugs. In adults with previous exposure to ARV drugs, resistance to first-generation INSTIs was 8.8% (95% CI: 6.6–11.5%). None of the people with previous ARV drug exposure and resistance to first-generation INSTIs reported receiving INSTI-based ART. The prevalence of the integrase mutation G163RK was 15.2% among adults without previous exposure to ARV drugs and 8.2% among those with previous exposure to ARV drugs (Table 4). This mutation was detected in all cases with resistance to first-generation INSTI. The cases in which G163KR was observed had infection with HIV-1 subtype F (34.6%), F1/B recombinants (34.6%), or other F recombinants (30.7%) (Table 2).

NNRTI-based ART regimens were the most frequently prescribed (55.1%, 95% CI: 54–56.1%), followed by dolutegravir (DTG)-based regimens (29.4%, 95% CI: 28.4–30.4%), and PI/r-based regimens (11.7%, 95% CI: 11.0–12.6%) (Table 1). Among people initiating and reinitiating NNRTI-based ART, 15.9% (95% CI: 14.4–17.5%) had resistance to EFV or NVP (Table 5). The prevalence of resistance to ATV/r or LPV/r was 4.8% among individuals initiating and reinitiating ATV/r- or LPV/r-based ART. Among those initiating and reinitiating DTG-based ART, none had DTG resistance.

**Figure 1:**
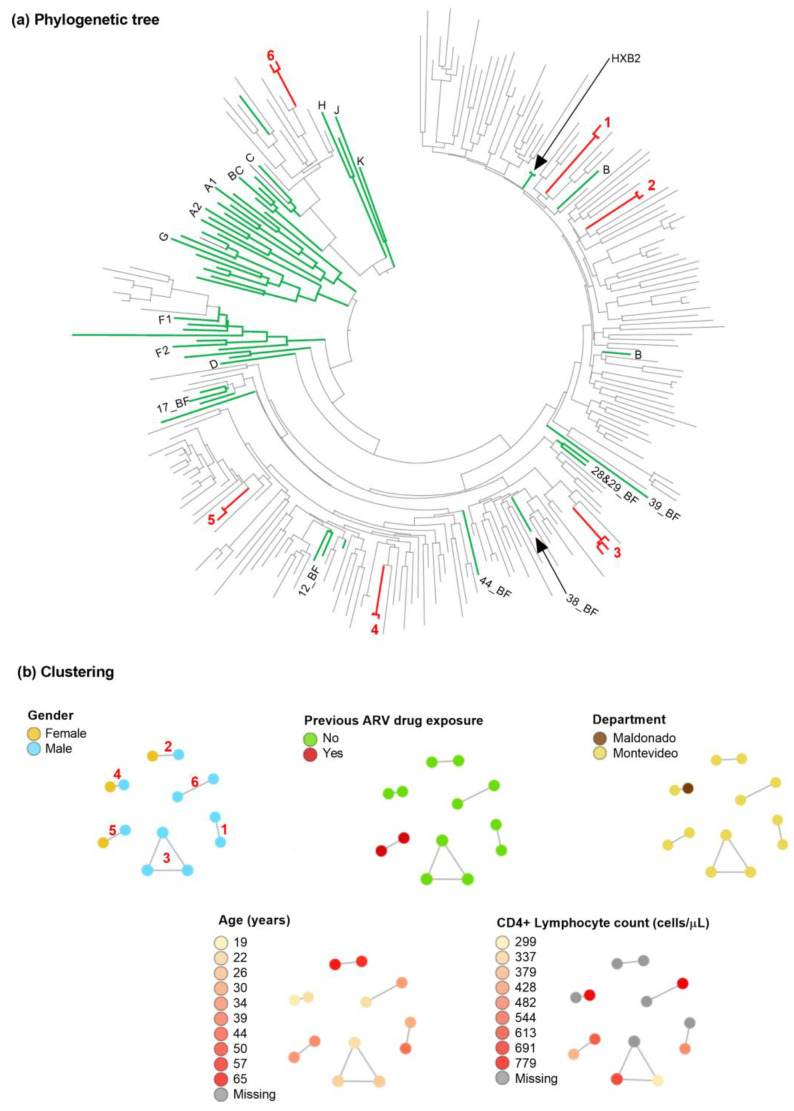
Phylogenetic tree and main clusters. (**a**) Phylogenetic tree. Sequences from 205 persons living with HIV participating in the HIV drug resistance survey in Uruguay were included with HIV-1 subtype references (green) obtained from Los Alamos HIV Database. Sequences were aligned with ClustalW. A maximum-likelihood tree was constructed with Mega v11, using the GTR + Г + I nucleotide substitution model with 1000 bootstrap repetitions. Possible clusters supported by bootstrap ≥99% are shown and numbed in the tree (red numbers 1–6). (**b**) Clustering. Putative transmission links were evaluated using HIVTRACE at a TN93 distance threshold of <1.5%. Only sequences in a cluster are shown. Nodes are coloured by different available attributes. The same clusters coloured in the tree are indicated in (**b**).

Concerning HIV transmission, six clusters of two to three individuals were observed (Figure 1). Five of the clusters corresponded to people without previous ARV drug exposure. Three groups had exclusively male individuals. Four clusters were from individuals residing in Montevideo, and one included individuals from Montevideo and Maldonado.

## 4. Discussion

In Uruguay, the prevalence of HIV drug resistance to EFV and NVP was high among people initiating and reinitiating ART. As reported in other Latin American countries [10,22,23,24,25,26], the observed prevalence was greater than 10%, underscoring the need to accelerate the transition to the WHO-recommended DTG-based first-line ART [27,28]. Uruguay included DTG in the national ART guidelines in 2018. Therefore, at the time of the survey, DTG-based regimens were available in the country, but only 29.4% of those initiating and reinitiating ART received these regimens.

The prevalence of HIV drug resistance to EFV or NVP was significantly higher among individuals with previous ARV drug exposure than those without ARV drug exposure. Therefore, access to DTG-based regimens should be prioritised for people reporting previous exposure to ARV drugs. In addition, retention in care must be improved [27,29], and adequate support for treatment adherence must be provided to prevent HIV drug resistance among people on ART, thus minimising the risk of transmission of HIV-resistant variants [27,29].

The resistance to the first-generation INSTI in Uruguay was attributed to the G163KR mutation, which is polymorphic in HIV-1 subtype F [15,16] and has been reported in high prevalence among BF recombinant virus circulating among INSTI-naive individuals [30]. Indeed, in Uruguay, the cases with G163KR mutation were INSTI-naive and had infection with subtype F, BF recombinants, or other F recombinants. The G163KR mutation has not been associated with HIV resistance to second-generation INSTIs. Therefore, an impact on WHO-recommended DTG-based ART is not expected.

Several clusters of HIV transmission were observed in this survey, including three clusters with male individuals only. WHO recommends oral pre-exposure prophylaxis (PrEP) as an additional prevention choice for HIV-negative adults at a high risk of HIV acquisition [27]. Recommended PrEP regimens contain TDF with or without FTC or 3TC [27]. In Uruguay, among people without previous exposure to ARV drugs, the prevalence of HIV drug resistance to TDF was low (3.1%), and resistance to FTC or 3TC was not observed. These results support using these ARV drugs for PrEP regimens in the country.

Finally, approximately one-third of adults initiating and reinitiating ART with CD4 lymphocyte count had less than 200 cells/µL. Therefore, it is relevant to strengthen the interventions for early HIV diagnosis, including self-testing, index testing, and assisted partner notification [31,32], timely linkage to treatment, and retention in care [27]. In addition, comprehensive management of advanced HIV disease should be provided at the healthcare facilities in the country [33].

A strength of the survey is that it was carried out following WHO recommendations, and the results can be considered nationally representative estimates of HIV drug resistance among adults initiating and reinitiating ART in Uruguay. A limitation was that previous exposure to ARV drugs was self-reported and may have been underreported. Another limitation was that the enrolment was extended beyond the planned 6-month period to achieve the sample size.

In conclusion, this survey generated relevant, actionable data to inform public health interventions in Uruguay. First, accelerating the transition to DTG-based first-line ART as the preferred option at the national level, and prioritising access to this regimen for people with previous exposure to ARV drugs, will be key to minimising the impact of HIV drug resistance on HIV epidemic control goals. Second, HIV drug resistance should not be considered a barrier to scale-up oral pre-exposure prophylaxis for HIV. Third, retention in care and adequate support for ART adherence must be maximised to prevent HIV drug resistance. Finally, interventions are needed to ensure early HIV diagnosis, timely linkage to treatment, and retention in care.

## Figures and Tables

**Table 1:** Demographic and clinical characteristics of adults living with HIV initiating and reinitiating antiretroviral therapy with and without previous exposure to ARV drugs in Uruguay, 2018–2019.

	All (N = 206)			Adults without Previous Exposure to ARV Drug (N = 133)			Adults with Previous Exposure to ARV Drugs (N = 73)		
	n	%	95% CI	n	%	95% CI	n	%	95% CI
Gender									
Women	71	34.3	33.2–35.4	34	25.5	24.3–26.8	37	50.2	47.7–52.7
Men	130	63.2	62.1–64.3	95	71.5	70.2–72.7	35	48.3	45.8–50.8
Transgender	4	2.0	1.6–2.5	3	2.3	1.8–2.9	1	1.5	0.8–2.7
Unknown	1	0.5	0.5–0.5	1	0.7	0.7–0.7	0	0.0	0.0–5.0
Age									
≤25 years	30	14.3	13.8–14.8	23	17.0	16.2–17.8	7	9.4	8.6–10.1
>25 years	176	85.7	85.2–86.2	110	83.0	82.2–83.8	66	90.6	89.9–91.4
Initiated first-line ART									
TDF + 3TC/FTC + EFV	84	41.0	39.6–42.3	72	54.7	53.3–56	12	16.1	14.9–17.4
TDF + 3TC/FTC + NVP	3	1.7	1.0–2.8	1	0.7	0.6–0.9	2	3.3	1.6–6.7
ZDV + 3TC/FTC + EFV	18	8.6	8.1–9.2	10	7.3	7.0–7.7	8	10.9	9.6–12.4
ZDV + 3TC/FTC + NVP	7	3.4	3.0–3.8	1	0.7	0.7–0.7	6	8.1	7.1–9.2
DTG-based	61	29.4	28.4–30.4	34	25.3	24.2–26.5	27	36.7	34.5–39.0
PI-based	24	11.7	11.0–12.6	8	6.0	5.3–6.8	16	22.1	20.1–24.3
Other	1	0.5	0.5–0.5	0	0.0	0.0–2.8	1	1.3	1.3–1.4
Unknown	8	3.8	3.6–3.9	7	5.1	4.9–5.4	1	1.3	1.3–1.4

Study design-weighted proportion and 95% confidence interval. INSTI-based regimens included dolutegravir or raltegravir. PI-based regimens included atazanavir/ritonavir, darunavir/ritonavir or lopinavir/ritonavir. ART: antiretroviral therapy; ARV: antiretroviral; CI: confidence interval; DTG: dolutegravir; EFV: efavirenz; NVP: nevirapine; PI: protease inhibitor; TDF: tenofovir disoproxil fumarate; ZDV: zidovudine; 3TC/FTC: lamivudine or emtricitabine.

**Table 2:** Proportional distribution of HIV subtypes in adults initiating and reinitiating ART in Uruguay, 2018–2019.

Subtype	All (N = 205)		With Integrase Resistance-Associated Mutation G163KR (N = 26)	
	n	%	n	%
HIV-1 subtype B	86	42.0	0	0.0
Recombinant of F1, B	52	25.4	9	34.6
HIV-1 subtype F1	17	8.3	9	34.6
Recombinant of 12_BF, B	13	6.3	3	11.5
HIV-1 subtype C	9	4.4	0	0.0
HIV-1 CRF 12_BF	6	2.9	3	11.5
Recombinant of B, A1	5	2.4	0	0.0
Recombinant of C, B	3	1.5	0	0.0
HIV-1 CRF 31_BC	3	1.5	0	0.0
Recombinant of 12_BF, A1	2	1.0	2	7.7
HIV-1 CRF 47_BF	2	1.0	0	0.0
HIV-1 CRF 20_BG	1	0.5	0	0.0
Recombinant of 31_BC, C	1	0.5	0	0.0
Recombinant of 42_BF, F1	1	0.5	0	0.0
Recombinant of B, 39_BF	1	0.5	0	0.0
Recombinant of B, 47_BF	1	0.5	0	0.0
Recombinant of B, C	1	0.5	0	0.0
HIV-1 CRF 42_BF	1	0.5	0	0.0

Unweighted proportions of HIV-1 subtypes assigned using the REGA HIV-1 subtyping tool (v3.46).

**Table 3:** Prevalence of HIV drug resistance among adults initiating and reinitiating ART with and without previous exposure to ARV drugs in Uruguay, 2018–2019.

	All		Adults without Previous Exposure to ARV Drug		Adults with Previous Exposure to ARV Drugs	
	n/N	%, 95% CI	n/N	%, 95% CI	n/N	%, 95% CI
NRTI						
Any	21/204	10.3, 9.4–11.2	11/131	8.5, 7.4–9.8	10/73	13.4, 12.5–14.4
ABC	12/204	5.9, 5.2–6.6	4/131	3.1, 2.4–4.1	8/73	10.7, 9.9–11.6
3TC or FTC	4/204	1.9, 1.7–2.2	0/131	0.0, 0.0–2.8	4/73	5.4, 4.8–6.0
TDF	7/204	3.5, 2.9–4.1	4/131	3.1, 2.4–4.1	3/73	4.0, 3.5–4.6
ZDV	16/204	7.9, 7.0–8.8	10/131	7.8, 6.6–9.1	6/73	8.0, 7.3–8.8
NNRTI						
EFV or NVP	31/204	15.2, 14.1–16.3	16/131	12.3, 11.0–13.8	15/73	20.3, 18.7–22.0
DOR	12/204	5.8, 5.3–6.2	4/131	2.9, 2.9–3.0	8/73	10.8, 9.7–12.0
EFV	30/204	14.7, 13.6–15.9	15/131	11.6, 10.2–13.0	15/73	20.3, 18.7–22.0
ETR	11/204	5.2, 4.9–5.6	6/131	4.5, 4.1–4.8	5/73	6.6, 6.1–7.2
NVP	31/204	15.2, 14.1–6.3	16/131	12.3, 11.0–13.8	15/73	20.3, 18.7–22.0
RPV	28/204	13.8, 12.6–15.2	18/131	14.1, 12.4–16.1	10/73	13.3, 12.5–14.2
PI/r						
ATV/r, DRV/r or LPV/r	3/204	1.5, 1.1–2.1	3/131	2.4, 1.7–3.2	0/73	0.0, 0.0–5.0
ATV/r	3/204	1.5, 1.1–2.1	3/131	2.4, 1.7–3.2	0/73	0.0, 0.0–5.0
DRV/r	1/204	0.6, 0.2–1.3	1/131	0.9, 0.4–2.1	0/73	0.0, 0.0–5.0
LPV/r	3/204	1.5, 1.1–2.1	3/131	2.4, 1.7–3.2	0/73	0.0, 0.0–5.0
INSTI						
Any	26/205	12.7, 11.7–13.8	20/132	14.9, 14.1–15.6	6/73	8.8, 6.6–11.5
BIC	0/205	0.0, 0.0–1.8	0/132	0.0, 0.0–2.8	0/73	0.0, 0.0–5.0
CAB	0/205	0.0, 0.0–1.8	0/132	0.0, 0.0–2.8	0/73	0.0, 0.0–5.0
DTG	0/205	0.0, 0.0–1.8	0/132	0.0, 0.0–2.8	0/73	0.0, 0.0–5.0
EVG	26/205	12.7, 11.7–13.8	20/132	14.9, 14.1–15.6	6/73	8.8, 6.6–11.5
RAL	26/205	12.7, 11.7–13.8	20/132	14.9, 14.1–15.6	6/73	8.8, 6.6–11.5

Study design-weighted proportion and 95% confidence interval. HIV drug resistance was defined as the presence of a penalty score ≥15 using the Stanford HIVdb algorithm. ABC: abacavir; ART: antiretroviral therapy; ATV/r: atazanavir/ritonavir; BIC: bictegravir; CAB: cabotegravir; CI: confidence interval; DOR: doravirine; DRV/r: darunavir/ritonavir; DTG: dolutegravir; EFV: efavirenz; ETR: etravirine; EVG: elvitegravir; FTC: emtricitabine; INSTI: integrase strand-transfer inhibitor; LPV/r: lopinavir/ritonavir; NNRTI: non-nucleoside reverse-transcriptase inhibitor; NRTI: nucleoside reverse-transcriptase inhibitor; NVP: nevirapine; PI/r: boosted protease inhibitor; RAL: raltegravir; RPV: rilpivirine; TDF: tenofovir disoproxil fumarate; ZDV: zidovudine; 3TC: lamivudine.

**Table 4:** Prevalence of HIV drug resistance-associated mutations among adults initiating and reinitiating antiretroviral therapy with and without previous exposure to ARV drugs in Uruguay, 2018–2019.

NRTI Resistance-Associated Mutations (%)			NNRTI Resistance-Associated Mutations (%)			PI resistance-Associated Mutations (%)			INSTI Resistance-Associated Mutations (%)		
Mutation	Adults without Previous Exposure to ARV Drugs (N = 131)	Adults with Previous Exposure to ARV Drugs (N = 73)	Mutation	Adults without Previous Exposure to ARV Drugs (N = 131)	Adults with Previous Exposure to ARV Drugs (N = 73)	Mutation	Adults without Previous Exposure to ARV Drugs (N = 131)	Adults with Previous Exposure to ARV Drugs (N = 73)	Mutation	Adults without Previous Exposure to ARV Drugs (N = 132)	Adults with Previous Exposure to ARV Drugs (N = 73)
M41L	2.3	2.7	L100IV	0.0	0.0	L23I	0.0	0.0	T66AIK	0.0	0.0
K65ENR	0.0	0.0	K101EHP	1.5	4.1	L24IFM	0.0	0.0	E92GQV	0.0	0.0
D67EGHNSTDel	0.8	1.4	K103HNST	7.6	15.1	D30N	0.0	0.0	G118R	0.0	0.0
T69DGDelIns	0.8	0.0	V106MA	0.0	0.0	V32I	0.8	0.0	F121CY	0.0	0.0
K70EGNQRST	0.0	0.0	V179FL	0.0	0.0	M46ILV	0.0	1.4	E138AKT	0.0	0.0
L74VI	0.0	1.4	Y181CFGISV	1.5	2.7	I47VA	0.8	0.0	G140ACRS	0.0	0.0
V75AIMST	0.0	0.0	Y188CFHL	0.0	1.4	G48LMQSTV	0.0	0.0	Y143CGHKRS	0.0	0.0
F77L	0.0	0.0	G190ACEQSTV	2.3	5.5	I50VL	0.0	0.0	S147G	0.0	0.0
Y115F	0.0	0.0	P225H	0.0	4.1	F53LY	0.0	0.0	Q148HKNR	0.0	0.0
F116Y	0.0	0.0	M230IL	0.0	0.0	I54VLMATS	0.8	0.0	N155HST	0.0	0.0
Q151LM	0.0	0.0	A98G	0.8	1.4	G73ADCSTV	0.0	0.0	S230R	0.0	0.0
M184VI	0.0	5.5	K103R	1.5	2.7	L76V	0.0	0.0	R263K	0.0	0.0
L210W	1.5	1.4	V106I	6.1	9.6	V82ATFSCML	1.5	0.0	H51Y	0.0	0.0
T215ACDEFILNSVY	6.1	6.9	V108I	1.5	1.4	N83D	0.0	0.0	Q95K	0.8	0.0
K219QENRW	0.8	2.7	E138A	6.9	6.9	I84VAC	0.0	0.0	T97A	3.0	0.0
E40F	0.0	0.0	E138GKQR	2.3	0.0	I85V	0.8	0.0	P145S	0.0	0.0
E44AD	0.0	0.0	V179DE	6.1	2.7	N88DGST	0.0	0.0	Q146P	0.0	0.0
A62V	1.5	1.4	H221Y	0.0	1.4	L90M	0.0	0.0	V151AL	0.0	0.0
S68Del	0.0	0.0	F227CILV	0.0	0.0	L10F	0.0	0.0	S153FY	0.0	0.0
			L234I	0.0	0.0	V11IL	1.5	0.0	E157Q	1.5	2.7
			P236L	0.0	0.0	K20T	0.0	0.0	G163KR	15.2	8.2
			K238NT	0.0	1.4	L33F	0.0	0.0	D232N	0.0	0.0
						K43T	0.0	0.0			
						Q58E	0.0	0.0			
						T74P	0.0	0.0			
						L89V	0.0	0.0			

Unweighted proportions of sequences with non-zero penalty scores in the Stanford HIVdb algorithm. Underscored mutations correspond to non-SDRMs (surveillance drug resistance mutations as defined in Bennett et al. [20] and Tzou et al. [21]) and may include polymorphisms. A total of 204 reverse transcriptase and protease sequences and 205 integrase sequences were available. NRTI: nucleoside reverse transcriptase inhibitors; NNRTI: non-nucleoside reverse transcriptase inhibitors; PI: protease inhibitors; INSTI: integrase strand transfer inhibitors.

**Table 5:** Prevalence of HIV drug resistance among adults initiating and reinitiating ART in Uruguay by regimen prescribed, 2018–2019.

	NNRTI-Based ART Prescribed		DTG-Based ART Prescribed		ATV/r- or LPV/r-Based ART Prescribed	
	n/N	%, 95% CI	n/N	%, 95% CI	n/N	%, 95% CI
NRTI						
Any	10/113	8.8, 8.0–9.7	9/59	15.0, 13.7–16.4	2/24	8.9, 5.6–13.9
ABC	4/113	3.5, 3.1–3.9	6/59	10.1, 8.9–11.4	2/24	8.9, 5.6–13.9
3TC or FTC	1/113	0.9, 0.7–1.1	3/59	5.0, 4.2–6.0	0/24	0.0, 0.0–13.8
TDF	2/113	1.7, 1.5–2.0	3/59	5.0, 4.2–6.0	2/24	8.9, 5.6–13.9
ZDV	7/113	6.2, 5.5–7.1	7/59	11.7, 10.5–12.9	2/24	8.9, 5.6–13.9
NNRTI						
EFV or NVP	18/113	15.9, 14.4–17.5	9/59	15.0, 13.7–16.3	3/24	13.3, 9.2–18.8
DOR	3/113	2.6, 2.4–2.8	7/59	11.6, 10.6–12.7	1/24	4.4, 2.4–8.1
EFV	17/113	15.0, 13.6–16.6	9/59	15.0, 13.7–16.3	3/24	13.3, 9.2–18.8
ETR	3/113	2.5, 2.5–2.6	7/59	11.6, 10.6–12.7	1/24	4.1, 3.3–5.1
NVP	18/113	15.9, 14.4–17.5	9/59	15.0, 13.7–16.3	3/24	13.3, 9.2–18.8
RPV	15/113	13.5, 11.6–15.7	9/59	15.0, 13.7–16.3	3/24	13.0, 9.4–17.7
PI/r						
ATV/r, DRV/r or LPV/r	1/113	0.8, 0.8–0.9	1/59	1.6, 1.6–1.7	1/24	4.8, 2.0–10.9
ATV/r	1/113	0.8, 0.8–0.9	1/59	1.6, 1.6–1.7	1/24	4.8, 2.0–10.9
DRV/r	0/113	0.0, 0.0–3.3	0/59	0.0, 0.0–6.1	1/24	4.8, 2.0–10.9
LPV/r	1/113	0.8, 0.8–0.9	1/59	1.6, 1.6–1.7	1/24	4.8, 2.0–10.9
INSTI						
Any	17/113	15.1, 13.4–16.9	7/60	11.7, 10.3–13.2	1/24	4.0, 3.7–4.3
BIC	0/113	0.0, 0.0–3.3	0/60	0.0, 0.0–6.0	0/24	0.0, 0.0–13.8
CAB	0/113	0.0, 0.0–3.3	0/60	0.0, 0.0–6.0	0/24	0.0, 0.0–13.8
DTG	0/113	0.0, 0.0–3.3	0/60	0.0, 0.0–6.0	0/24	0.0, 0.0–13.8
EVG	17/113	15.1, 13.4–16.9	7/60	11.7, 10.3–13.2	1/24	4.0, 3.7–4.3
RAL	17/113	15.1, 13.4–16.9	7/60	11.7, 10.3–13.2	1/24	4.0, 3.7–4.3

Study design-weighted proportion and 95% confidence interval. HIV drug resistance was defined as the presence of a penalty score ≥15 using the Stanford HIVdb algorithm. ABC: abacavir; ART: antiretroviral therapy; ATV/r: atazanavir/ritonavir; BIC: bictegravir; CAB: cabotegravir; CI: confidence interval; DOR: doravirine; DRV/r: darunavir/ritonavir; DTG: dolutegravir; EFV: efavirenz; ETR: etravirine; EVG: elvitegravir; FTC: emtricitabine; INSTI: integrase strand-transfer inhibitor; LPV/r: lopinavir/ritonavir; NNRTI: non-nucleoside reverse-transcriptase inhibitor; NRTI: nucleoside reverse-transcriptase inhibitor; NVP: nevirapine; PI/r: boosted protease inhibitor; RAL: raltegravir; RPV: rilpivirine; TDF: tenofovir disoproxil fumarate; ZDV: zidovudine; 3TC: lamivudine.

## Data Availability

The data presented in this study are available on request from the corresponding author.
